# Nucleus accumbens corticotropin-releasing factor increases cue-triggered motivation for sucrose reward: paradoxical positive incentive effects in stress?

**DOI:** 10.1186/1741-7007-4-8

**Published:** 2006-04-13

**Authors:** Susana Peciña, Jay Schulkin, Kent C Berridge

**Affiliations:** 1 Department of Psychology, University of Michigan, Ann Arbor, MI 48109, USA; 2 Department of Physiology and Biophysics, Georgetown University, CNE Branch, National Institute of Mental Health, USA

## Abstract

**Background:**

Corticotropin-releasing factor (CRF) is typically considered to mediate aversive aspects of stress, fear and anxiety. However, CRF release in the brain is also elicited by natural rewards and incentive cues, raising the possibility that some CRF systems in the brain mediate an independent function of positive incentive motivation, such as amplifying incentive salience. Here we asked whether activation of a limbic CRF subsystem magnifies the increase in positive motivation for reward elicited by incentive cues previously associated with that reward, in a way that might exacerbate cue-triggered binge pursuit of food or other incentives? We assessed the impact of CRF microinjections into the medial shell of nucleus accumbens using a pure incentive version of Pavlovian-Instrumental transfer, a measure specifically sensitive to the incentive salience of reward cues (which it separates from influences of aversive stress, stress reduction, frustration and other traditional explanations for stress-increased behavior). Rats were first trained to press one of two levers to obtain sucrose pellets, and then separately conditioned to associate a Pavlovian cue with free sucrose pellets. On test days, rats received microinjections of vehicle, CRF (250 or 500 ng/0.2 μl) or amphetamine (20 μg/0.2 μl). Lever pressing was assessed in the presence or absence of the Pavlovian cues during a half-hour test.

**Results:**

Microinjections of the highest dose of CRF (500 ng) or amphetamine (20 μg) selectively enhanced the ability of Pavlovian reward cues to trigger phasic peaks of increased instrumental performance for a sucrose reward, each peak lasting a minute or so before decaying after the cue. Lever pressing was not enhanced by CRF microinjections in the baseline absence of the Pavlovian cue or during the presentation without a cue, showing that the CRF enhancement could not be explained as a result of generalized motor arousal, frustration or stress, or by persistent attempts to ameliorate aversive states.

**Conclusion:**

We conclude that CRF in nucleus accumbens shell amplifies positive motivation for cued rewards, in particular by magnifying incentive salience that is attributed to Pavlovian cues previously associated with those rewards. CRF-induced magnification of incentive salience provides a novel explanation as to why stress may produce cue-triggered bursts of binge eating, drug addiction relapse, or other excessive pursuits of rewards.

## Background

Corticotropin-releasing factor (CRF) is involved in mediating the physiological and behavioral responses to stress [1-5]. Beyond activating aversive behaviors, stress also increases some appetitive behaviors. For example, food intake and relapse into drug-taking in models of addiction are both increased by natural CRF-releasing stressors [6-11]. Such appetitive behaviors have often been viewed as attempts to reduce or avoid unpleasant stress or as overflow from general motor arousal or frustration ([12-14] but see [8-11]).

However, despite the traditional association of CRF with aversive stress, some CRF brain systems also are activated by positive rewards, even in the absence of conventional stressors. For example, CRF release is increased in the central nucleus of the amygdala by spontaneous food ingestion [10,15] and in prefrontal cortex by presentation of Pavlovian cues associated with food reward [10]. Similarly, sucrose ingestion normalizes CRF levels in hypothalamus and amygdala in adrenalectomized rats [16]. In addition, CRF has also been suggested to play an important role in drug reward. For example, cocaine administration also activates CRF expression in CRH-producing neurons in hypothalamus and amygdala [13] and CRF given intracerebroventricularly (ICV) or into the ventrolateral BNST can induce reinstatement of drug self-administration behavior in the absence of external stressors (though this last could reflect attempts to escape aversive CRF effects) [17].

Reward activation of CRF raises the possibility at least that some brain CRF/glucocorticoid systems may participate in positively valenced processes of incentive motivation for reward. One candidate for positive motivation is the attribution of incentive salience to conditioned stimuli that predict rewards, magnification of which causes cue-triggered phasic peaks of increased incentive motivation for associated rewards. Incentive salience (i.e., cue-triggered 'wanting' for a reward, but not necessarily 'liking' for the same reward) has been shown to depend in part on ascending dopamine projections to nucleus accumbens [8-11]. Other neurochemical signals converging on the nucleus accumbens from mesocorticolimbic circuits could play roles in enhancing incentive salience too, either directly or by via dopamine modulation [18-20]. Does a CRF subsystem in nucleus accumbens participate in magnifying the incentive salience of cues previously associated with reward? If so, that provides a novel explanation for why stress sometimes potentiates appetitive motivated behavior, especially when reward cues are encountered. An incentive salience contribution would serve as alternative or supplement to traditional explanations such as escape from aversive states (i.e., anxiety, irritability, dysphoria) or general arousal [12,13,21]. An incentive-salience mechanism could also be relevant to understanding relationships between stress and neural sensitization in causing drug abuse, especially cue-triggered relapse into addiction [22].

Here we tested the specific hypothesis that elevated CRF neurotransmission in nucleus accumbens shell enhances the incentive salience of cues that have been associated with sucrose reward, and so magnifies 'wanting' for sucrose. Nucleus accumbens contains levels of CRF receptors that are comparable to those in amygdala [23-25]. The medial shell of nucleus accumbens also contains substantial distributions of CRF-immunoreactive and fibers, which might arise in part from hypothalamic CRF systems linked to stress, as well as cell bodies (particularly in its caudal half, and thus we focused on caudal shell here) [26]. Whether CRF acts directly on nucleus accumbens circuits in ways comparable to dopamine, or interacts with dopamine release in nucleus accumbens, CRF release in nucleus accumbens might facilitate incentive salience mechanisms [27-31]. Taken together with previous findings regarding the importance of nucleus accumbens in incentive salience amplifications by amphetamine and sensitization, it seems likely that if stress does modulate incentive salience via CRF mechanisms it might do so here in medial shell [1-5,30,31].

To selectively measure the enhancement of incentive salience by CRF, we used a pure incentive version of the Pavlovian-Instrumental transfer paradigm. This procedure measures the intensity of peaks of incentive motivation that are triggered by reward cues (a phenomenon that has been called cue-triggered 'wanting'). It excludes non-incentive explanations related to stress or stress reduction as well as other alternative explanations for increased behavior (e.g., primary instrumental response reinforcement, secondary response reinforcement, discriminative stimulus occasion setting, general stress/frustration drive, Pavlovian S-R habits) [29-37]. In short, features of the pure incentive Pavlovian-Instrumental transfer (PIT) procedure allow isolation of incentive salience enhancement by CRF from other stress-related mechanisms as explanation for magnified, sudden and intense cue-triggered peaks in incentive motivation for reward.

Our results suggest that activation of CRF systems in the medial accumbens shell can indeed enhance incentive salience attributed to reward cues. Incentive salience, amplified by CRF in nucleus accumbens, provides a new explanation of why stressors sometimes cause increased pursuit of rewards, especially at moments when stressed individuals encounter cues that were previously associated with those rewards.

## Results

### CRF and amphetamine in nucleus accumbens increase cue-triggered lever pressing associated with sucrose reward

**Figure 1:**
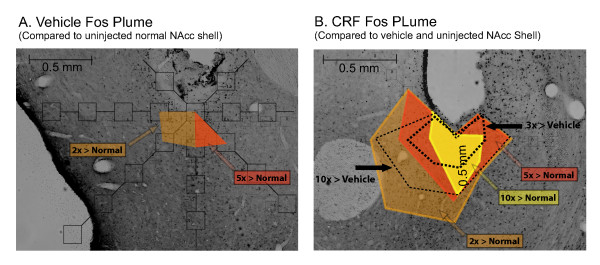
**Fos plumes**. Coronal sections showing point sample positions used to identify local Fos plumes around microinjection site. A. Vehicle microinjection produces merely low elevation of Fos compared to normal virgin tissue. Radial arms extending from center show sampling points for Fos measurement (125 μm × 125 μm blocks; 5× magnification). B. CRF microinjection induces intense elevation over normal tissue levels (depicted by color: 10× elevation over normal denoted by yellow, 5× = dark orange; 2× = light orange). CRF also causes elevation compared to vehicle microinjection levels at equivalent points (depicted by dotted lines; 3× relative increase over vehicle-levels denoted by thick dotted line, 5× = thin dotted line; upper right; plume from CRF 500 ng dose in 0.2 μl microinjection volume).

**Figure 2:**
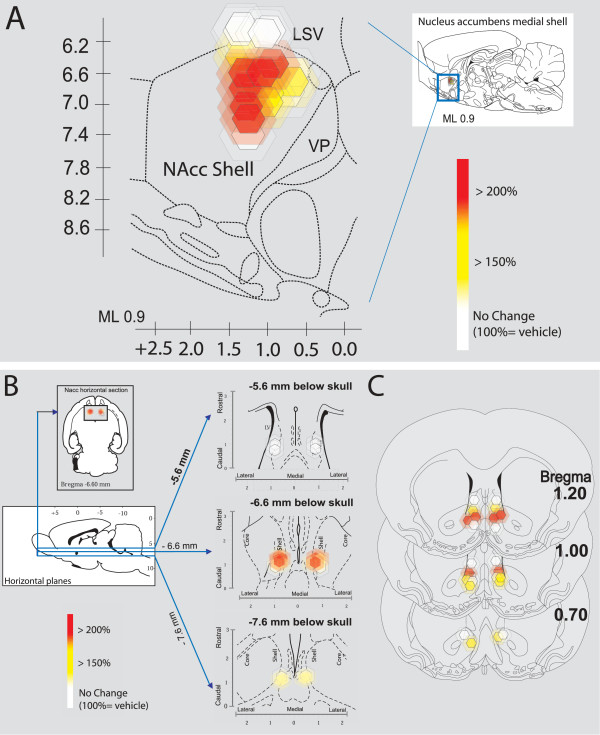
**Fos plume maps of CRF (500 ng) amplification of cue-triggered increases in lever pressing**. Fos plume maps of functional localization: CRF effects on cue-triggered incentive salience. Sagittal (A), horizontal (B), and coronal maps (C) show each Fos plume as colored symbol in medial shell of nucleus accumbens. Color depicts magnification effect of CRF (500 ng) microinjection at that site on peaks of lever pressing triggered by a 30 sec auditory CS+ previously associated with sucrose reward (within-subject percentage elevation of CRF versus vehicle in the same rat; 100%=vehicle). Size of central symbol depicts radius of intense Fos elevation; size of surrounding halos depict outer radius of moderate Fos elevation. For the sagittal map, bilateral accumbens sites from left and right sides of each rat brain are collapsed together into one combined sagittal map of accumbens for simplicity. Maps adapted from Paxinos and Watson [46].

**Figure 3:**
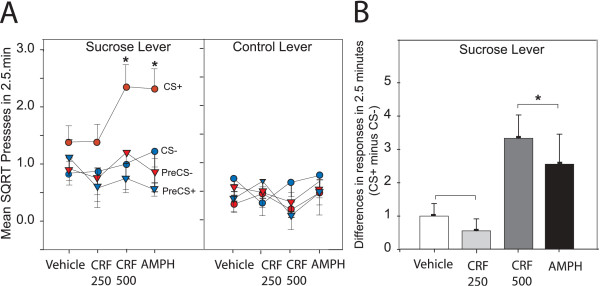
**Enhancement of incentive motivation by CRF**. Effects on cue-triggered lever pressing during extinction test caused by CRF (250 and 500 ng) and amphetamine (20 μg) microinjections in the caudal medial accumbens shell. A. Lines show pressing in the presence of the CS+ (previously associated with sucrose- red circles); and CS- (never associated with sucrose- blue circles); and pre-CS+ baseline period (no-stimulus 30 sec period immediately prior to CS+ stimulus); and pre-CS- baseline period (30 sec immediately prior to CS- stimulus). The left panel depicts lever pressing on the control lever that had previously produced instrumental sucrose reward during training (but not during extinction test). The right panel depicts lever pressing on the control lever that was never had produced reward. Both 500 ng CRF and 20 μg amphetamine increased the ability of the sucrose CS+ cue (but not CS-) to trigger enhanced responding on the previously sucrose-associated lever (but not on the control lever). B. Transform scores show a direct contrast between CS+ and CS- effects of lever pressing. CS+ effects on cue-triggered lever pressing are amplified by CRF (500 ng) and by amphetamine (20 μg) microinjections.

CRF (high dose 500 ng/0.2 μl; low dose 250 ng/0.2 μl) and amphetamine (20 μg/0.2 μl) microinjections were located in the region of medial shell of nucleus accumbens that contains highest CRF levels (Figure 1; Figure 2). CRF and amphetamine each dramatically and similarly increased cue triggered incentive motivation to obtain reward (Three-way ANOVA (drug × cue presence × lever), F_3,159 _= 5.57; p < 0.05, main effect of drug). Both CRF and amphetamine comparably increased phasic CS+-triggered peaks of increased pressing on the lever that previously had been associated with sucrose reward compared to vehicle, but did not increase lever pressing at other times (i.e., during CS- or in the absence of any CS) (Two-way ANOVA (drug × cue presence), F_3,79 _= 4.21; p < 0.05, significant interaction) (Figure 3).

Post-hoc comparisons indicated that CRF enhancement of cue-triggered peaks of lever pressing was exclusively due to the highest dose of CRF (500 ng; Bonferroni, p < 0.05). Specifically, 500 ng CRF microinjections tripled the amplitude of phasic peaks in pressing on the sucrose-associated lever during the 30-sec CS+ tone or clicker and in the 30-sec period immediately after it, compared to the same lever after vehicle microinjections (Figure 3), but not during other periods. An equivalent tripling of cue-triggered peaks in lever pressing was also produced by amphetamine (20 μg) microinjections at the same sites (Bonferroni, p < 0.05) (Figure 3), used as a standard here for enhanced cue-triggered 'wanting', and confirming previous results [30,31]. By contrast, the low dose of CRF (250 ng) failed to significantly enhance active lever pressing over vehicle despite the possibility of a slight trend (Bonferroni, p = 0.42, n.s), and by itself the 30-sec CS+ elicited merely a modest rise of only 50% over pre-cue baseline in lever pressing after vehicle microinjections, which was less than half the maximum drug-enhanced levels.

### Associative cue specificity: CS+ vs baseline

In order to rule out motor arousal and general frustration effects of CRF as explanations of increased peaks in incentive motivation, it is crucial that enhancement not also apply to the plateaus of lever-pressing during periods between cue-triggered peaks. Motor arousal and aversive effects can be caused by intracerebral microinjections CRF [38-40]. However, motor arousal and frustration effects of CRF would be expected to remain relatively constant over a 30 min test, or at least last longer than the minute or so impact of a cue. Thus if motor arousal or aversive states were the explanation for elevated instrumental behavior here, lever pressing during baseline periods in between auditory stimuli should have been elevated too – not only when a CS+ was present. However, neither CRF (500 or 250 ng) nor amphetamine (20 μg) produced detectable increases in baseline instrumental responding during no-stimulus baseline periods (One-way ANOVA (drug), F_3,39 _= 0.3, p = 0.8, n.s.). Baseline instrumental responding in the absence of any CS (i.e., pre-cue periods) was never increased by any microinjection (even when it increased lever pressing in response to the CS+). This dissociation between enhanced cue-triggered incentive motivation during CS+ versus unchanged baseline responding was separately confirmed in a two-way ANOVA by a significant interaction between the presence/absence of CS+ and administration of either CRF (drug × cue presence interaction, F_2,119 _= 5.23, p < 0.05) or amphetamine (F_1,79 _= 4.25, p < 0.05) (Figure 3).

### Associative cue specificity: CS+ vs. CS-

Similarly, the CS+ (tone or click) was the only conditioned stimulus that ever significantly increased lever pressing on the sucrose associated lever (Three-way ANOVA (cue type × drug × lever), F_1,119 _= 10.40, p < 0.001, main effect of cue type; Figure 3). The control CS- (a different auditory stimulus that did not predict sucrose reward) failed to increase lever pressing over the baseline pre-cue period in any drug condition (Figure 3). That selective pattern of only-CS+ enhancement is required by the hypothesis of incentive salience magnification, which posits that enhanced incentive salience is attributed specifically to stimuli previously associated with reward.

A useful way of further assessing the associative specificity of CS+ effects versus CS- effects on Pavlovian-Instrumental transfer effects is to calculate transfer scores between them. A transfer score directly contrasts pressing during CS+ versus CS- by subtracting CS- pressing from CS+ pressing on a within-subject basis, so that the remainder shows the enhanced CS+ effect for each individual rat. When we calculated this, the resulting transfer scores confirmed that both the CRF high dose (500 ng) and amphetamine (20 μg) microinjections significantly and specifically amplified the incentive effect of the CS+ on pressing the sucrose-associated lever, compared to vehicle and low dose CRF microinjections (Figure 3).

Thus, CRF (500 ng) and amphetamine (20 μg) magnification of cue-triggered peaks in lever pressing applied only to the CS+ that previously was associated with sucrose UCS, and not to the control CS- which did not signal sucrose.

### Temporal reversibility of cue-triggered pursuit: dependence on CS+ presence

**Figure 4:**
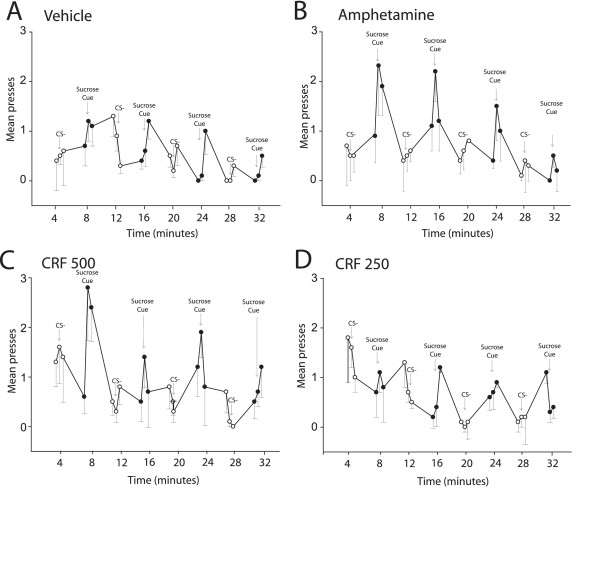
**Temporal pattern of lever pressing within test session**. Minute-by-minute time-course of lever pressing peaks on sucrose-associated lever after microinjections of (A) vehicle, (B) amphetamine 20 μg, (C) CRF 500 ng, and (D) CRF 250 ng. Open circles depict lever pressing during presentation of the 30-sec CS- and filled circles during presentation of the CS+. Lever pressing is also shown during the 30 sec period before each CS+ or CS- (precue baseline), and during the 30 sec period that followed each CS+ or CS- (post-cue period). Both CRF (500 ng) and amphetamine (20 μg) magnified intense, transient, reversible, and repeatable peaks in pressing on sucrose-associated lever each time the CS+ occurred. Magnified lever pressing after CRF (500 ng) or amphetamine (20 μg) was triggered by each presentation of the sucrose cue, but then was followed by a rapid descent back to normal baseline levels of pressing once the cue ended (B and C).

If increased lever pressing for reward is caused by excessive attribution of incentive salience to the reward's CS+, then the temporal pattern of peaks of cue-triggered responding should be phasic, reversible, and repeatable [30,31]. In other words, peak bursts of active lever pressing should come and go with successive presentations of the 30-sec CS+, and disappear again soon after each presentation. A temporal analysis of responding confirmed this pattern for CRF (500 ng) and amphetamine (20 μg) enhancement of pressing on the sucrose-associated lever during CS+ presentations (Figure 4). A magnified peak of pressing on the lever previously associated with sucrose reward was triggered anew by each presentation of the CS+ sucrose cue after CRF (500) or amphetamine microinjections, maximally elevating during its 30-sec period (Two-way ANOVA (cue presence × cue presentation order), F_1,79 _= 16.11, p < 0.001; main effect of cue presence). Pressing rose within seconds of each CS+ and remained dramatically elevated throughout the 30 sec presentation. After almost every CS+, pressing decayed significantly within 1 min of its end, and always descended to baseline levels again before the next CS occurred 4 min later.

This temporal pattern of cue-triggered phasic peaks of pressing (without enhancing intervening plateaus or CS- pressing) is important because it further rules out alternative explanations mentioned above, based on traditional CRF/amphetamine effects that have more constant time course durations (i.e., on the order of several minutes or more). Those include increased motor arousal, general impulsiveness or aversive states that would last longer than 30 sec, and so should be present during no-stimulus baseline periods (and CS- presentations) between CS+ tones. By contrast, the enhanced incentive salience interpretation specifies that CRF should specifically amplify attribution only to the reward CS+, just as amphetamine microinjection does, and so seems to fit best this observed pattern of effects.

### Target specificity of cue-triggered incentive motivation: active versus inactive levers

Cue-triggered increases in efforts to obtain sucrose were directed specifically to the active lever that had previously earned sucrose reward during training, and not to the other lever (Two-way ANOVA (drug × lever), F_3,79 _= 3.25; p < 0.05, significant interaction; Figure 4). Rats made few responses on the second control lever that had never been associated with sucrose reward. Pressing on the control lever was not increased during the CS+ by either CRF (500 ng) (Two-way ANOVA (drug × cue presence), F_2,59 _= 0.35, p = 0.55, n.s.) or amphetamine (20 μg) (Two-way ANOVA (drug × cue presence), F_2,59 _= 0.36, p = 0.55, n.s.). Lever specificity was further confirmed by finding a significant difference between previously-active lever versus always-inactive lever in pressing during CS+ (Two-way ANOVA, F_1,159 _= 5.12, p < 0.05), and a significant interaction between lever identity and drug in the microinjection-induced enhancement of cue triggered increases in pressing (Two-way ANOVA (drug × lever), F_1,159 _= 34.15; p < 0.001). Thus, the CS+ sucrose cue selectively triggered increased pressing only on the lever previously associated with obtaining sucrose. That suggests that our CRF incentive on PIT lever pressing are not explained by nonspecific motor arousal induced by CRF, or a general sensorimotor tendency to emit more pressing movements regardless of target, even if CRF produced any motor or arousal effects (Figure 4).

### Pavlovian approach CRs to sucrose dish

**Figure 5:**
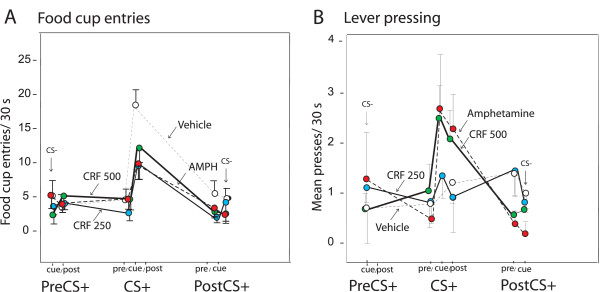
**Response competition between sucrose dish approach and lever pressing**. A. Averaged time-course of approach responses to sucrose dish just before, during and after first CS+ cue presentation. B. Same information for pressing on sucrose-associated lever. Each panel compares cue-triggered responding after microinjections of vehicle (white circles), CRF 500 μg (green circles), CRF 250 μg (blue circles), or amphetamine 20 μg (red circles). Both CRF and amphetamine reduced the peak of cue-triggered approaches to the sucrose dish, but simultaneously increased cue-triggered lever pressing. It is impossible to perform both actions at exactly the same time, and so conditioned dish approaches may have been suppressed as a result of response competition from increased lever pressing that occurred during the same 30-sec period.

Presentations of CS+ also elicited Pavlovian approach conditioned responses to the sucrose dish (especially after vehicle microinjections), either as a Pavlovian S-R habit elicited by the CS+, or as a discriminative instrumental response to earn sucrose. However, CRF and amphetamine microinjections both suppressed conditioned dish approach below the vehicle control level (F_3,159 _= 2.79, p < 0.05; Figure 5). That suppression is noteworthy because it helps rule out the several remaining alternative explanations. For example, approach suppression by CRF indicates that the enhancement of PIT cannot be due to CRF potentiation of S-R Pavlovian habits, because dish approach was the only S-R habit ever actually paired with CS+ tone during training. Similarly, approach suppression indicates that CRF did not potentiate discriminative instrumental responding (in which the CS+ might have acted as a discriminative stimulus (S_D_) to signal that instrumental approach would be reinforced, and this instrumental relation might later have been generalized to lever pressing [41,42] because any such instrumental relation between approach and reinforcement was actually suppressed by CRF and amphetamine during the PIT test, and not enhanced. Sucrose dish entries during the first CS+ were especially suppressed by both CRF doses (Two-way ANOVA (drug × cue presence), F_2,79 _= 4.21, p < 0.01). Sucrose dish approaches were similarly suppressed by amphetamine microinjection (F_1,39 _= 6.40, p < 0.05). Suppression of dish approach during the CS+ by CRF and amphetamine may have occurred possibly as a secondary consequence of increased response competition from higher cue-triggered rates of lever pressing that occurred at the same time.

The suppressive effects on dish approach CRs after microinjection of CRF or amphetamine were associatively specific to the CS+ (drug × CS+ interaction, F_1,39 _= 6.40, p < 0.05) (Fig. 3), and the same was true for amphetamine microinjection (drug × CS+ interaction, F_1,39 _= 5.34, p < 0.05). Neither CRF nor amphetamine microinjections decreased food cup entries below vehicle levels during the baseline 30 s pre-cue periods (2-way ANOVA, significant interaction (drug × cue presence), F_3,79 _= 3.47, p < 0.05) or during CS- presentations (drug × CS+ interaction, F_1,39 _= 3,25, p = 0.68, n.s.).

Finally, we note that CRF effects on PIT versus dish approach were independent and partially dissociable by dose: both 250 ng and 500 ng CRF doses suppressed cue-triggered dish approach during CS+ presentations early in a session, but only 500 ng CRF increased lever pressing. That indicates that suppression of dish approach is not a sufficient cause of CRF enhancement for cue-triggered lever pressing, which appears to require independent incentive motivation mechanisms recruited by the higher dose.

### Fos plumes: identifying zones of local neuronal activation

To help identify where our CRF microinjections actually acted in the brain we used a Fos plume mapping procedure to visualize spread of neuronal gene transcription triggered by 500 ng CRF in tissue around microinjection sites [43-45]. The local expression of Fos protein is a useful marker for geographic spread of pharmacological impact, even if c-fos transcription is not invariably tied to neuronal activation, at least for drugs that induce acute Fos transcription immediately around a microinjection site (including CRF). Measuring the size of the resulting local Fos plume caused by a microinjection objectively assesses the spherical volume of tissue and intensity of neuronal response impacted by local drug action. Fos expression in a local neuron might either reflect direct drug action on receptors on that neuron, or else reflect indirect action from adjacent neurons containing receptors that act on Fos-expression neurons via local circuits. In either case, the Fos plume reflects a local sphere of functional modulation induced by CRF microinjection, and provides quantitative information on its intensity and size. The boundaries of the plume reveal where the drug stops having an intense functional impact, even if drug molecules spread further beyond the plume in lower concentrations insufficient to trigger gene transcription.

For microinjections of CRF (500 ng) we identified two zones of elevated Fos expression: intense versus moderate elevation zones (Figure 1). Each zone was defined by 2 independent criteria. An inner zone of intense Fos elevation was defined as the mean radius within which 1) absolute Fos expression was increased by at least 1 order of magnitude over normal tissue levels (10 times spontaneous levels in medial shell), and 2) relative Fos expression was increased by at least twice over the levels produced at equivalent locations after vehicle microinjections (2 times vehicle-induced levels). An outer zone of moderate Fos elevation was defined at the mean radius within which 1) absolute Fos expression was increased by 3 times (but not 10 times) over normal tissue levels, and 2) relative Fos expression was increased by 5 times over the level of equivalent points after vehicle locations (note: the reason why our outer zone used a higher vehicle-relative threshold than the inner zone was that a vehicle microinjection induces some Fos expression in a small centrally-restricted inner zone, perhaps due to pressure of the microinjection or irritation from cannula-induced damage. The resulting slight elevation in inner zone vehicle baseline [above normal tissue baseline] imposed a ceiling on drug-induced Fos expression in this central zone that limited relative increases to under 5× [even if drug caused a >10× absolute increase]).

The mean inner zone of intense Fos elevation produced by CRF microinjection at the behaviorally most effective dose (500 ng) was approximately 0.25 mm in radius (absolute 10× increase over normal; relative 2× increase over vehicle) and the outer zone of low elevation was an additional 0.2 mm radius (absolute 5× increase over normal; relative 5× increase over vehicle; Figure 1). Therefore, to represent these zones we assigned color-coded symbols of corresponding 0.25 mm radius in size to represent each inner zone, surrounded by similarly colored but more pale halos of additional 0.2 mm radius to represent each outer zone. Thus symbols and halos served to bracket the estimated range of intense-moderate functional activation likely to mediate the mapped microinjection effects on behavior.

To calculate the 3-dimensional plume volume produced by CRF 500 ng microinjections, we assumed a roughly spherical shape and calculated the inner intense sphere to contain a volume of approximately 0.06 mm^3^, and the outer sphere to contain a volume of approximately 0.38 mm^3^. Because the entire medial shell is approximately 2.87 mm^3 ^in total volume, these volumes meant that the inner sphere of a CRF (500 ng) plume filled approximately 2% of total medial shell volume, whereas its outer sphere filled approximately 13%. Of course, some microinjections near borders may not have filled the shell to quite that extent if they partially penetrated other structures such as the medial core (though adjacent penetration was never deeper than 0.5 mm).

We mapped the extent of activation diffusion as reflected by Fos plume around each microinjection site. In order to further assess the role of diffusion into other structures or the ventricles, we took two additional steps. First, we compared PIT effects for 'hits' in nucleus accumbens shell versus 'missed' anatomical control sites in lateral septum dorsal to the nucleus accumbens. No enhancement of PIT by CRF or amphetamine was evident in rats with dorsal anatomical control sites outside the nucleus accumbens (Figure 5), indicating that the PIT enhancement effects described above was not due to diffusion upwards along cannulae tracks to dorsal structures. Second, we inspected individual brain slices to identify any sites in medial shell where the Fos plume could touch the wall of the lateral ventricle. This seemed worth doing because intra-ventricular CRF administration in the 1000 ng range has been reported to produce arousal and conditioned aversion effects, and so conceivably might have been responsible also for PIT magnification effects here. To assess if leakage into ventricles was the primary source of CRF effects here, we compared magnitude of PIT enhancement by CRF in rats with Fos plumes that touched the ventricle wall versus the group of rats with plumes contained entirely within the nucleus accumbens. Two rats had plumes that definitely touched lateral ventricle, and another two had plumes that might have touched, whereas 6 rats had plumes fully contained away from the ventricle wall. By themselves, the group of 4 rats with ventricle-touching plumes did not show a significant PIT enhancement effect by CRF (mean score 2.7, SEM = 1.3; F_1,7 _= 2.03, p = 0.20). By contrast, the group with non-touching plumes contained fully within nucleus accumbens away did show an enhancement of PIT by CRF microinjection (mean score 3.33; SEM = 1.17; F_1,63 _= 10,17, p < 0.05). That pattern across groups indicates that the significant PIT enhancement effects described above were not driven primarily by CRF ventricle diffusion in this group. Finally, we found no significant difference between these two groups (F_1,9 _= 0.10, p = 0.75), indicating that most sites tested in nucleus accumbens contributed comparably to the PIT effects described above.

### Functional Fos plume mapping of microinjection effects on behavior

Functional site effects were mapped for all cannulae placements in nucleus accumbens and adjacent structures (Figure 2). Colors for symbols and halos represented the magnitude of behavioral effects produced by CRF (500 ng) microinjections at each microinjection site change score in elevation of pressing on sucrose-associated lever minus to the control vehicle effect at that same site in the same rat). We chose the sagittal plane primarily to map Fos plumes and functions because sagittal view allows the entire rostrocaudal and dorsoventral extents of medial shell to be viewed on a single atlas map (Figure 2). Additional supplemental maps were also constructed in coronal and horizontal planes to allow full 3-D mapping of functional effects [46].

The Fos plume maps for localization of function showed that most nucleus accumbens microinjections were successfully placed in the intended posterior-central zone of medial shell that has highest levels of CRF receptors and terminals, and within that target zone most CRF (500 ng) microinjections produced comparable magnifications of PIT (Figure 2). The spread of local Fos plumes did not otherwise extend to other structures around the medial shell, including dorsal structures, except that a few microinjections may have penetrated approximately 0.4 mm into the medial border of nucleus accumbens core.

Although more remains to be done in future regarding localization of CRF function for incentive motivation in nucleus accumbens and related structures, these initial maps indicate that CRF activation in our present study was restricted to the nucleus accumbens medial shell, and possibly a small strip of adjacent core. Within that zone of medial shell, CRF activation appears sufficient to magnify the level of incentive salience attributed to a CS+ that was previously associated with reward (Figure 2).

## Discussion

Our findings show that a CRF subsystem in nucleus accumbens can magnify the positive incentive motivation triggered by a cue previously associated with reward (CS+), and so spur appetitive behavioral pursuit of that reward. CRF (500 ng) microinjections in medial shell of nucleus accumbens directly increased cue-triggered instrumental lever pressing behavior aimed at that sucrose reward, without increasing behavior in the absence of the Pavlovian CS+ or reward cue. The CRF-induced pattern of excessive cue-triggered pursuit of reward appeared remarkably similar to that caused by amphetamine microinjection in the same rats at the same sites [30,31], suggesting that both CRF and amphetamine in the nucleus accumbens may prime cue-triggered seeking of incentives via overlapping mesolimbic mechanisms. This is the first specific evidence to support the hypothesis that a brain limbic CRF sub-system can magnify any purely positive or appetitive motivational process, such as increased incentive salience, to spur cue-triggered pursuit of rewards [10].

### Perceptual synergy of cue with CRF/amphetamine incentive salience attribution

Enhancement of instrumental lever pressing co-depended on the simultaneous presence of CRF (or amphetamine) microinjection in nucleus accumbens plus the Pavlovian auditory cue in the environment that previously was associated with sucrose reward (Pavlovian CS+). Neither CRF nor cue alone was sufficient to produce maximum peaks of incentive motivation. Each time the CS+ was presented to a rat after CRF microinjection its lever pressing was dramatically magnified to a peak roughly double the level shown by the same rat before and after that CS+ (i.e., compared to either during absence of any CS [baseline] or during the CS- that did not predict sucrose reward), and roughly 300% over normal peaks that rat showed during the same CS+ on tests after control vehicle microinjections. Each cue-triggered peak lasted approximately 1 min, and instrumental responding then always fell back to control levels (Figure 3).

This selective pattern of enhancement limited to CS+ indicates that motivation enhancement was not a tonic consequence of CRF effects that lasted minutes or more (e.g., due to motor arousal or aversive state effects). Instead cue-triggered incentive motivation came and went with each CS+ as an intense time-locked phasic, reversible and repeatable peak, fitting the prediction of the hypothesis that CRF in nucleus accumbens can magnify the incentive salience of cues associated with reward, rather than directly driving increased levels of appetitive behavior in the absence of reward cues. This reversible CRF pattern is highly similar to the cue-locked enhancement pattern of amphetamine observed here and in previous experiments, a pattern that originally helped implicate identify incentive salience features mediated by mesolimbic dopamine systems [30,31].

### Ruling out alternative explanations

#### Not hedonic self-medication or reinforcement?

The ability of stress to promote pursuit of rewards is in a sense over-explained by traditional interpretations. That is, there are many different potential alternative explanations already available that may apply to some cases of real-world stress (e.g., stress self-medication, aversion escape, frustration or general arousal), and even more alternatives introduced in various animal models of triggered-relapse (e.g., discriminative instrumental S_D _signaling of reward availability). The potential over-abundance of explanations makes it especially important to rule out traditional ones before positing any new explanation, such as the hypothesis that CRF in nucleus accumbens magnifies an appetitive or positive motivation mechanisms such as cue-triggered incentive salience. So it is worth briefly summarizing how features of CRF results in the pure incentive PIT procedure used here eliminate traditional alternatives that might otherwise have explained CRF's amplification of cue-triggered incentive motivation for reward.

A common view of how stress might motivate binge eating and food intake, addictive drug relapse, gambling, or other excessive reward pursuit is to induce anhedonia, dysphoria, frustration, or related negative aversive states that individuals try to counteract by pursuing and consuming hedonic rewards [21,47]. CRF may indeed produce aversive states at many brain sites. However, several observations rule out aversion escape or mood enhancement by reward consumption as explanations for CRF's enhancement of cue-triggered incentive motivation for sucrose. First, rats never obtained any primary sucrose reinforcement while under CRF effects, and so no sucrose consumption ever actually improved their hedonic state during test. Further, amphetamine microinjection, which is generally thought to produce positive motivational effects via mesolimbic dopamine and related neurotransmitter activation, produced exactly the same pattern of enhanced cue-triggered incentive motivation as CRF. Most important, if CRF caused a negative state that maintained increased seeking of sucrose, then that pharmacological state should have remained visible throughout most of the half-hour test. Again, however, tonic elevation was not expressed in lever pressing; instead elevated peaks of lever pressing came and went with the auditory CS+ stimulus under CRF. Finally, it is relevant to note that the CS+ could not have acted as an instrumental discriminative stimulus to signal sudden sucrose availability, because our rats had never in their lives been instrumentally reinforced for pressing the lever in the presence of the CS+ (not even during training), and instead the only behavioral response that had ever earned sucrose before was actually reduced during CS+ by CRF (dish approach). Overall, therefore, it is not plausible that CRF caused rats to press more as an instrumental attempt to escape or reduce a persistent negative hedonic state.

#### Not sensorimotor arousal?

Several of the same considerations also rule out other alternative explanations for our results such as CRF-induced arousal, psychostimulant or accelerant, or general sensorimotor activity effects. For example, although CRF in the nucleus accumbens does indeed cause increased locomotion and grooming behavior [48], that chronic state-like consequence would apply over most of the entire 30-min test regardless of whether CS+ was present or absent. But baseline pressing was not elevated in the absence of CS+, and that cue-triggered pattern of the sucrose-lever increase, together with the lack of increase on the inactive lever, seem to rule out elevated general motor arousal as explanation for CRF's ability to magnify cue-triggered peaks of incentive motivation

#### Interaction with other CRF and limbic systems

We emphasize that our finding that CRF in medial shell of nucleus accumbens causes increased incentive salience attribution to reward CS+ does not contradict traditional notions that CRF systems in other brain structures may mediate mostly negative or aversive stress effects. Nor does it contradict suggestions that some appetitive behavior seen in stress may reflect attempts to reduce those aversive effects. However, it shows that the alternative phenomenon of enhanced positive incentive motivation triggered by reward-related cues may co-occur when generated by some CRF subsystems, such as in the medial shell of nucleus accumbens. This conclusion implies that blocking aversive effects per se may not be enough to block the increased in appetitive pursuits produced by stress. Instead, the increase in cue-triggered pursuit of incentives follows rules that govern the attribution of incentive salience to Pavlovian conditioned stimuli for reward [37,49].

The similar enhancements by CRF and amphetamine of cue triggered incentive motivation indicates it might be of future interest to examine potential interactions between CRF and mesolimbic dopamine or corticolimbic glutamate projections in modulating reward cue signals in nucleus accumbens. Interaction is compatible with reports that, for instance, aversive stressors can cause dopamine release in nucleus accumbens (which has sometimes been used to argue against a purely positive motivational role of dopamine) [50,51]. Regardless of the role of dopamine per se in stress, CRF might interact with mesolimbic mechanisms of incentive salience in nucleus accumbens either directly or by modulating dopamine-related and/or glutamate-related signals [52]. We caution that, although potential interaction might possibly seem to imply that CRF antagonists could be used therapeutically to reduce excessive incentive salience in stress, the actual effect of CRF receptor manipulation on dopamine release in nucleus accumbens may involve complexities that could complicate such predictions. For example, the CRF-1 antagonist CP-154,526 has been reported to increase cocaine-associated dopamine release, at least in rostral shell of nucleus accumbens and pre-frontal cortex [53], and so any speculation about the potential relation of CRF antagonists to incentive salience must await future empirical investigation.

## Conclusion

### Paradoxical positive incentive motivation magnification by CRF systems in nucleus accumbens?

Everyone agrees that stress is predominantly an aversive motivational state. Similarly, CRF delivered to hypothalamus and amygdala may produce predominantly aversive effects, which typically suppress appetitive behaviors such as normal food intake [54]. However, our findings indicate that particular CRF subsystems, particularly in nucleus accumbens, magnify a positive motivational process triggered by reward cues that independently spurs pursuit of reward. Specifically, our results that CRF receptor activation in medial shell of nucleus accumbens directly increase the attribution of incentive salience to a CS+ that was previously associated with reward. Phasic cue-triggered peaks of incentive motivation to obtain sucrose reward are seen whenever a synergistic combination of conditions is met: simultaneous CS+ or cue presence plus CRF co-activation in nucleus accumbens. CRF's magnification of cue-triggered incentive salience appears remarkably similar to effects of amphetamine in nucleus accumbens, suggesting that stress and addictive drugs might both prime excessive cue-triggered seeking of incentives via overlapping mesocorticolimbic mechanisms.

One possibility for a natural role is that CRF nucleus accumbens systems might ordinarily be activated in conjunction with stress-related glucocorticoid elevation. That seems consistent with demonstrations that HPA activation and glucocorticoid hormones may contribute to binge eating, drug addiction, and other excessive reward pursuits [22,55]. Glucocorticoids actually increase CRF expression in structures such as amygdala and BNST, and conceivably in accumbens shell too [56,57]. Thus, stress and glucocorticoids might 'energize' goal directed behaviors in part by activating CRF accumbens mechanisms that amplify the incentive salience of stimuli associated with reward [56,58]. Future studies will be needed to address the question of how CRF interacts with glucocorticoids, dopamine, and other limbic neurochemical systems to modulate incentive salience.

Our results suggest that although stress and hypothalamic CRF have predominantly aversive motivational effects, activation of CRF systems in the medial accumbens shell can actually enhance appetitive behavior via purely positive motivational effects. This provides a novel explanation for why stress may sometimes exacerbate cue-triggered binge eating, drug addiction relapse, and other excessive pursuits of rewards.

## Methods

### Subjects

Experimentally naïve male Sprague Dawley rats (n = 23), (born at the University of Michigan; 230–260 gm at the beginning of the experiment) were housed in pairs in plastic tub cages under a reverse 12 hr light cycle (lights off at 7:00 a.m.). Rats were divided into two groups that received identical surgery for implantation of microinjection cannulae; one group (n = 14) was used for behavioral training and testing, cresyl violet histology and ink cannulae localization, and the other group (n = 9) was used for Fos plume analyses under microinjection conditions identical to the first behavioral test day. Rats were given 15–20 gm of rat chow each day during the experiment and tap water was provided *ad libitum *in the home cage. All studies were approved by the University Committee on Use and Care of Animals of the University of Michigan in compliance with National Institutes of Health standards.

### Test chambers

Training and testing took place in computerized operant chambers (Med Associates Inc., St. Albans, VT). Each chamber contained a sucrose cup (with photobeam entry detector), two levers, and speaker modules (clicker and tone). A 3 W, 24 V house light mounted on the top-center of the wall opposite the magazine provided illumination. Sound attenuating boxes equipped with ventilation fans masked external noise. A computer equipped with MED-PC software (Med Associates) recorded the number of active and inactive lever presses and food cup entries. A videocamera positioned below the transparent floor recorded the rat's behavior for subsequent slow-motion analysis.

### Behavioral measure of incentive salience motivation

Phasic peaks in incentive salience attributed to reward that were triggered by sudden presentations of an associated CS+ (cue-triggered incentive motivation) were measured in a pure conditioned incentive paradigm based on Pavlovian-Instrumental transfer (PIT). It is important to note that PIT procedures by themselves are not pure measures of incentive salience, as PIT effects contain both motivational and specific UCS-signaling information components. Instead the procedures must be carefully designed to conform to pure incentive rules and exclude alternative interpretations (e.g., test in extinction, preserve instrumental baseline unchanged, etc). Our pure incentive PIT has been designed to do just that.

### Instrumental training (days 1–17)

We used a variable interval schedule (with ascending levels of responding demand) for instrumental training to produce low but stable instrumental responding in the face of extinction that would optimally reveal the motivational form of Pavlovian instrumental transfer relevant to incentive salience. Rats were initially given overnight sucrose pellets twice in their home cages to familiarize them with the reward and overcome neophobia (days 1 and 2). All animals received next two sessions of magazine training (days 3 and 4) in which 20 deliveries of a single 45 mg sucrose pellet (Formula F; P.J. Noyes Co., Lancaster, PA) were freely given on a fixed time (FT), 1 min schedule of reinforcement. Then animals were shifted to a variable interval (VI)-1 instrumental reinforcement schedule (each reward was delivered on average every 5 seconds after at least one lever press) (days 5–7). Presses on one lever in the operant chamber produced sucrose pellets, whereas presses on the other lever did not, and served only as control for sensorimotor effects. Active and inactive lever assignments were balanced across rats. The schedule of reinforcement for the active lever was progressively increased to VI-5 (days 8–11), VI-10 (days 12–14) and VI-30 (days 15–17).

### Pavlovian training (days 18–31)

Pavlovian conditioning took place immediately after instrumental training. Both levers were absent from the chambers during Pavlovian conditioning to prevent adventitious reinforcement of a lever pressing habit during the cue, and to eliminate possibilities of pairing the CS+ with a lever pressing habit. Auditory stimuli each lasting 30 sec were arbitrarily designated as CS+ and CS-: an auditory clicker stimulus (60 dbs) and a pulsed tone stimulus (2.9 kHz, 60 dbs, continuous pulsing 0.5 sec on/off). During 14 daily sessions, rats received 10 pairings of the CS+ (30 sec duration) followed by immediate delivery of three sucrose pellets [unconditioned stimulus (UCS)]. Similar to previous PIT studies, we wished the CS- to serve as a control stimulus that was essentially irrelevant to reward and rewarded performance, rather than to suppress instrumental pressing as would either a novel stimulus or a strongly negative stimulus that was extensively paired with reward omission [59]. Therefore the CS- (30 sec duration) was presented twice during the last three Pavlovian sessions (at the middle and end of each session) and was never followed by sucrose pellets [29]. Nose entries into the sucrose dish were monitored by photobeam detector, and all rats developed a discriminative approach conditioned response (CR) to the sucrose dish during the CS+ but not during the CS- (CR difference scores were calculated to verify acquisition as approaches during 30 sec CS minus approaches during 30 sec baseline immediately before that CS).

### Microinjection cannula surgery

Rats were pretreated with 0.1 ml of atropine sulfate, anesthetized with a mixture of ketamine HCL (80 mg/kg, i.p.) and xylazine (5 mg/kg) and stereotaxically implanted with bilateral 23 gauge guide cannulae targeted at the medial shell of the nucleus accumbens. A slanted skull position was used, with the incisor bar set at +5 mm above interaural zero to achieve a slanted cannula angle and avoid penetrating the lateral ventricles. Cannulae were aimed at the caudal half of the accumbens: 2.8-2.2 mm anterior to bregma, 0.9 mm lateral to the midline, and 5.7 mm ventral to the skull surface (2.0 mm above the injection site), based on the atlas of Paxinos and Watson (1996). These coordinates corresponded to the following coordinates with a flat skull: 1.2-0.7 mm from bregma, 0.9 mm from midline and 7.0 mm from the skull. The cannulae were anchored with skull screws and cranial cement, and wire stylets were used to prevent cannula occlusion. Animals were allowed to recover for seven days before testing.

### Final lever retraining and extinction pre-exposure (days 32 and 33)

Before testing, rats were given two additional instrumental training sessions to re-establish instrumental lever pressing (VI-30 sec schedule), followed by two instrumental extinction days (days 34 and 35) with levers present but no sucrose to habituate animals to the extinction condition.

### Drugs and microinjections

Bilateral microinjections of vehicle (sterile isotonic saline) or corticotropin releasing factor (human CRF, 250 and 500 ng/0.2 μl per side; Sigma St. Louis, MO) were selected based on previous CRF microinjection studies. For example, 500 ng in the nucleus accumbens increased activation general behavioral activity counts, whereas 125 or 250 ng did not (and neither dose did when administered in lateral ventricles, consistent with an accumbens site of action) [32].

D-amphetamine sulfate (20 μg/0.2 μl per side; Sigma St. Louis, MO) has been previously found to increase cue-triggered incentive motivation in this pure conditioned incentive (PIT) paradigm after microinjections into nucleus accumbens shell [24 and 25] and was used as a 'gold standard' for increased CS+ incentive salience to compare against CRF effects within each rat. CRF 250 and 500 ng doses were chosen because they have previously been found to produce grooming, sniffing and oral motor behavioral effects when microinjected in nucleus accumbens shell [48]. Microinjections were made with a stainless steel injector cannula (29 gauge), extending 2.0 mm beyond the ventral tip of the guide, and attached to a syringe pump via PE-20 tubing. Rats were gently hand-held during the bilateral microinjections (0.2 μl at a rate of 0.30 μl/min via syringe pump). After infusion, the injectors remained in place for an additional 60 sec to allow for drug diffusion before the obturators were replaced.

### Conditioned incentive testing of cue-triggered incentive motivation (days 36–42)

On each of 4 test days rats were given bilateral microinjections of either vehicle (0.2 μl), CRF (250 or 500 ng/0.2 μl) or amphetamine (20 μg/0.2 μl) and placed back in their cages. The order of microinjection was counterbalanced across rats, and test days were spaced 48 hrs apart. After 15 min (approximately when behavioral effects such as CRF-induced grooming or amphetamine-induced locomotion began to be observed), rats were placed in the instrumental chambers with both levers present (days 36, 38, 40 and 42) for a 32.5 min test session for Pavlovian-Instrumental transfer). Instrumental performance was assessed under extinction conditions, so no sucrose pellets were given at any time during the test. In each test session, the CS+ and CS- were each presented four times for 30 sec each presentation (first CS+ or CS- presented at 2.5 min; fixed time 4 min interval; alternating CS+/CS- order). Presses on both previously-active and previously-inactive levers were recorded automatically during each 30 sec CS+ and CS- presentation, during the 30 sec period immediately preceding each CS+/CS- (baseline), and during the 30 sec period immediately after each cue presentation. Sucrose dish entries were also recorded during these periods.

### Statistical analysis

Each rat served as its own control for within-subjects ANOVA comparison of drug and CS effects on instrumental lever pressing responses and on dish approach Pavlovian CRs (vehicle vs drugs across test days; CS+ vs baseline vs CS- within test session). Lever pressing totals during each period were square root transformed (SQRT) to achieve homogeneity of variance as assessed by the Mauchly Sphericity test prior to ANOVA. Differences scores for cue-triggered effects of each CS were obtained by subtracting the number of responses during each baseline precue period from responses during its subsequent CS+ or CS- presentation [29]. Within subject three-way ANOVAs were performed to examine the effects of lever (active vs nonactive), cue drug microinjection (vehicle, CRF 250 and 500 ng) and discriminative cue type (CS+ vs. CS-). The effects of drug administration on baseline lever pressing (30 s precue time period) was also assessed, as was the order of cue presentation within a cue session (first, second, third or fourth), using 2-way ANOVAs. Whenever main effects or interactions were found, the Bonferroni method was used for *post hoc *comparisons. Similar analyses were performed for entries into the sucrose dish.

### Histology

Following the completion of testing, rats were deeply anesthetized with sodium pentobarbital, microinjected either with CRF (500 ng) or ink and 1-hr later perfused transcardially with buffered saline, followed by a buffered 4% paraformaldehyde solution. The brains were removed, post-fixed, sectioned (40 μm), and mounted on slides. Brain slices were prepared for one of 2 analyses: 1) densities and diameters were measured for local Fos plumes caused by microinjections of CRF or vehicle in a group of rats not used for behavioral tests; 2) microinjection centers were mapped by ink microinjection and cresyl violet staining in all rats used for behavioral testing. Two animals were found to have placements located outside of the nucleus accumbens shell and so were considered separately to yield a final total of 10 subjects with accurate placements in medial shell.

### Fos-like protein immunohistochemistry

Fos plumes were measured in rats that were microinjected bilaterally in medial shell of nucleus accumbens with the most behaviorally effective doses of 500 ng or vehicle. Seventy-five minutes after microinjection, rats were deeply anesthetized with sodium pentobarbital prior to transcardial perfusion. Brains were removed and placed in 4% formaldehyde for 2 hours, placed in 30% sucrose overnight, and then sectioned at 50 μm and stored in 0.2 M NaPb (pH 7.4). To visualize Fos-like immunoreactivity, we used the avidin-biotin procedure [60]. Brain sections were immersed in blocking solution (3% normal goal serum (NGS) and 0.3% Triton X-100 in TPBS) for 1 h and then incubated at room temperature for 24 h with a rabbit polyclonal antiserum directed against the N-terminal region of the Fos gene (Sigma, St Louis; dilution of 1:5000 in TPBS, 1% NGS and 0.3% Triton X-100). To reduce background staining the antiserum was preabsorbed with acetone-dried rat liver powder overnight at 4°C. After the primary antibody incubation, tissue was exposed to goat anti-rabbit, biotinylated secondary IgG (Santa Cruz Biochemicals, California; diluted 1:200) and then to avidin-biotin-peroxidase complex for 1 h at room temperature. A nickel diaminobenzidine (Nickel-DAB) glucose oxidase reaction was used to visualize Fos-like immunoreactive cells. Finally, sections were washed in Tris buffer, mounted from ddH2O, air-dried, dehydrated in alcohol, cleared in xylene and coverslipped. Fos-like immunoreactivity was visualized using a Leica microscope coupled to a SPOT RT slider (Diagnostic Instruments, MI) using SPOT software (SPOT version 3.3).

### Fos plume identification

Our procedure for measuring Fos plumes immediately surrounding a local microinjection site that are induced by drug was modified slightly from one previously described [43-45]. Briefly, Fos-labeled cells on tissue surface with 5x–40x magnification were counted individually within blocks (125 μm × 125 μm) at point locations spaced at 125 μm intervals along each of 7 radial arms emanating from the center of the microinjection site (45°, 90°, 135°, 180°, 225°, 270°, 315°; Figure 1).

Fos densities were measured 1) in normal nucleus accumbens medial shell tissue of brains without a microinjection cannula to assess normal baseline expression, 2) around the site of vehicle microinjections to assess cannula track and vehicle-induced Fos baseline expression, and 3) around the site of CRF microinjections to assess drug-induced elevations of local Fos expression (Figure 1).

Fos plumes surrounding drug microinjections were mapped as zones of intense or moderate elevation of Fos expression, identified in two ways: 1) as absolute increases over medial shell normal levels (elevation by 10 times [intense] or 5 times [moderate] normal tissue counts sampled in the absence of any cannula track, and 2) as vehicle-relative increases caused by drug (elevation by 5 times or 2 times over vehicle microinjection-induced levels at equivalent point locations around drug vs. vehicle microinjection tracks) (Figure 1).

### Mapping procedure of microinjection plumes for localization of function

Cannulae used for behavioral tests after microinjections were first located by measuring how far ventral ink extended directly below a microinjection cannula, and identifying the midway point on the vertical line between the bottom of the cannula and the ventral edge of the ink 180° below. This center point was localized within a 0.1 mm margin of error and plotted on the coronal atlas page that best fit that slice. The dorsal-ventral and medial-lateral coordinates were read off the coronal atlas axes (+0.1 mm accuracy; [46] and the anterior-posterior coordinate of the point was taken either from the coronal atlas page (if the slice fit the page closely) or from the midpoint between two adjacent atlas pages (if the slice appeared intermediate between atlas sections; +0.2 mm accuracy). Initial placements were made in the coronal plane because the coronal plane has been most thoroughly mapped in available standard atlases, and its greater degree of detail facilitated more precise mapping of site centers. The 3 coordinates of the center point were then transformed into sagittal atlas locations to provide a single simultaneous view of all microinjection sites in medial shell of nucleus accumbens (accurate to 0.01 to 0.2 mm). Coronal and horizontal maps were also constructed to provide full 3-dimensional information on the location of Fos plume spheres.

## Authors' contributions

SP conceived and designed the study, carried out behavioral testing and statistical analysis, participated in interpretation and drafted the manuscript; JS co-conceived the study and participated in writing the manuscript; KCB conceived the framework for the study, participated in design and interpretation and helped draft and rewrite the manuscript.
